# Home Endotoxin Exposure and Wheeze in Infants: Correction for Bias Due to Exposure Measurement Error

**DOI:** 10.1289/ehp.7981

**Published:** 2005-08-12

**Authors:** Nora Horick, Edie Weller, Donald K. Milton, Diane R. Gold, Ruifeng Li, Donna Spiegelman

**Affiliations:** 1 Department of Biostatistics, and; 2 Department of Environmental Health, Harvard School of Public Health, Boston, Massachusetts, USA; 3 Channing Laboratory, Harvard Medical School, Boston, Massachusetts, USA; 4 Department of Epidemiology, Harvard School of Public Health, Boston, Massachusetts, USA

**Keywords:** asthma, endotoxin, measurement error, regression calibration, wheeze

## Abstract

Exposure to elevated levels of endotoxin in family-room dust was previously observed to be significantly associated with increased wheeze in the first year of life among a cohort of 404 children in the Boston, Massachusetts, metropolitan area. However, it is likely that family-room dust endotoxin was a surrogate for airborne endotoxin exposure. Therefore, a related substudy characterized the relationship between levels of airborne household endotoxin and the level of endotoxin present in house dust, in addition to identifying other significant predictors of airborne endotoxin in the home. We now reexamine the relationship between endotoxin exposure and wheeze under the assumption that the level of airborne endotoxin in the home is the exposure of interest and that the amount of endotoxin in household dust is a surrogate for this exposure. We applied a measurement error correction technique, using all available data to estimate the effect of endotoxin exposure in terms of airborne concentration and accounting for the measurement error induced by using house-dust endotoxin as a surrogate measure in the portion of the data in which airborne endotoxin could not be directly measured. After adjusting for confounding by lower respiratory infection status and race/ethnicity, endotoxin exposure was found to be significantly associated with a nearly 6-fold increase in prevalence of wheeze for a one interquartile range increase in airborne endotoxin (95% confidence interval, 1.2–26) among the 360 children in households with dust endotoxin levels between the 5th and 95th percentiles.

Bacterial endotoxin is a lipopolysaccharide found in the outer cell membrane of gram-negative bacteria (GNB). Among its many known biologic activities, endotoxin is a cause of airway inflammation when inhaled. Exposure to endotoxin is associated with increased risk of nonatopic wheeze and with reduced prevalence of inhalant allergy, eczema, and atopic wheezing. Given the pervasive presence of GNB in household dust and air, everyone is exposed to at least low levels of environmental endotoxin.

In the past decade, studies have linked endotoxin in house dust with increased severity of asthma in both adults (Michel et al. 1991, 1996) and children (Rizzo et al. 1997). Recently, Park et al. (2001a) demonstrated that, after controlling for cockroach allergen, lower respiratory illness, smoking during pregnancy, lower birth weight, maternal asthma, presence of dog, and race/ethnicity, exposure to an elevated level of endotoxin in settled family-room house dust [≥100 endotoxin units (EU)/mg] during the first year of life is associated with a marginally significant increased risk of “any wheeze” [relative risk (RR) = 1.33; 95% confidence interval (CI), 1.00–1.76] and a significantly increased risk of “repeated wheeze” (RR = 1.56; 95% CI, 1.03–2.38). This study used endotoxin measurements obtained from 404 living-room floor-dust samples to quantify the presence of endotoxin, and dichotomized individual exposure into high and low categories using the sample median as the cutoff.

To what extent, however, does the amount of endotoxin present in settled family-room house dust reflect individual exposure? Although ingestion of dust endotoxin is a possible route of exposure for infants and toddlers, it is likely that the relevant exposure for irritant airway symptoms is inhaled (airborne) endotoxin. Therefore, it may be argued that the amount of endotoxin present in a household’s air rather than in the settled dust better characterizes its inhabitants’ exposure to endotoxin. If this is the case, then studies of airway irritant symptoms such as the one conducted by Park et al. (2001a) have used a surrogate measure to quantify the relationship between exposure and disease, while employing statistical methods that assume the exposure is perfectly measured. It should be noted that because endotoxin levels in air are determined by many factors in addition to the amounts of endotoxin present in settled dust, direct measurement of airborne endotoxin may give a truer indication of exposure than do dust measurements, even though dust measurements may integrate exposure over longer periods of time.

Our aim in the present analysis is to reexamine the relationship between exposure to endotoxin and wheeze in the first year of life, accounting for the measurement error associated with using house-dust endotoxin measurements as surrogates for true exposure. To this end, using dust endotoxin and wheeze outcome measurements from a large longitudinal study and airborne endotoxin measurements from a validation substudy, we performed a measurement error correction analysis according to the regression calibration method described by Rosner et al. (1989). With this method, the bias incurred by using a surrogate exposure metric, which controls random error, is removed; the resulting effect estimate is given in terms of the hypothesized “true” exposure metric, and the variance of this estimate reflects the fact that exposure is estimated among those without airborne endotoxin measurements.

## Materials and Methods

The Epidemiology of Home Allergens and Asthma study is an ongoing longitudinal study conducted among 499 families in the Boston, Massachusetts, metropolitan area. This study includes children born between September 1994 and June 1996 in Brigham and Women’s Hospital and having at least one parent with a history of doctor-diagnosed allergy and/or asthma. The first visit to the homes of participating families occurred 2–3 months after birth of the index child, at which time samples of dust from the living-room floor were collected in a standardized fashion (Park et al. 2001a) and analyzed using the kinetic Limulus assay with resistant-parallel-line estimation (KLARE) (Milton et al. 1992, 1997). There were 404 living-room samples with sufficient dust to perform an endotoxin assay. In addition to the home visits when dust collection took place, caregivers were contacted by telephone on a monthly basis and asked about their child’s respiratory health, including their wheezing. Information from those monthly phone calls made during the index child’s first year was summarized to reflect whether each child experienced “any wheeze,” corresponding to one or more wheezing events in the first year. Thus, the main study potentially could consist of those 404 families who contributed both wheeze outcomes and living-room floor endotoxin measurements.

Living-room airborne endotoxin measurements were collected among a 23% subset (*n* = 93) of these 404 main study families and assayed for endotoxin using the KLARE method as previously described (Park et al. 2001b). Samples were collected on 0.4-μm polycarbonate filters at 2 L/min for an average of 1.5 days. In 36 homes, airborne measurements were obtained concurrently with dust measurements collected at the time of the first home visit, whereas in the remaining 57 homes, airborne samples were collected during a subsequent visit that occurred 6–8 months after the birth of the index child. The airborne measurements obtained during the latter home visits do not accurately reflect the child’s exposure to endotoxin before assessment of the outcome and thus cannot be used for exposure–response modeling. Therefore, only those 36 families who contributed airborne samples during the first home visit are considered members of the internal validation study, and the 57 families contributing airborne samples at the 6- to 8-month visit comprise the external validation study. Five homes contributed airborne samples on two occasions, the latter of which was not considered in this analysis, bringing the total number of homes in the validation study to 88. After excluding participants missing data on key model covariates, the final validation study sample size was 82.

### Statistical methods.

Assay of floor-dust samples is a common approach in assessing allergen exposure in environmental studies. However, the degree to which such measures accurately reflect pulmonary endotoxin exposure is not entirely clear. When considering airways irritation, the amount of endotoxin suspended in the air, rather than the amount present in settled family-room dust, may represent a more valid gauge of endotoxin exposure, because the route of exposure is respiratory. Therefore, we identified airborne endotoxin as the true exposure and house-dust endotoxin as the surrogate exposure in the present analysis, recognizing that repeated air sampling over the course of the year and personal rather than area sampling could have provided a still more accurate measure of exposure than that considered in this study. As long as the variation in air endotoxin samples varies randomly around individual’s true long-term average exposure, as we believe to be the case here, the methods applied in this article will be valid (Spiegelman et al. 1997; Wacholder et al. 1993).

Rosner et al. (1989) proposed a regression calibration method for correcting odds ratio and corresponding CI estimates for systematic and random measurement error using a logistic regression model. InAppendix 1, we briefly show the applicability of this technique for obtaining measurement-error–corrected estimates of prevalence and risk ratios, which we directly estimate here, because it is well known that for a common event such as wheeze, the odds ratio overestimates the risk and prevalence ratios (Skov et al. 1998; Wacholder 1986; Zocchetti et al. 1997). The derivation given inAppendix 1 also applies to RR estimators obtained by Poisson regression with the robust variance, which can be used validly and, often, with little loss of efficiency when there are numerical difficulties fitting the log-binomial model (Spiegelman and Hertzmark 2005; Zou 2004).

Rosner’s method requires a main study/ validation study design, in which outcome and continuous surrogate exposure measurements are obtained in *n*_1_ main study participants, and true and surrogate exposures are measured in *n*_2_ validation study participants. The RR regression model fit in the main study expresses the (log-transformed) probability of the binary outcome as a linear function of the surrogate exposure and other covariates assumed to be measured without error, and is estimated using the main study data. The resulting estimate of risk ( 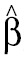) represents the (log) RR of outcome associated with a one-unit increase in the surrogate exposure level, uncorrected for measurement error. When the log-binomial model fails to provide the maximum likelihood estimates because of convergence or other numerical problems, one may obtain a generalized estimating equation (GEE) estimator by fitting a robust log-Poisson model (Zou 2004). Constructed using data from the validation study, the measurement error model expresses the true exposure as a linear function of the surrogate exposure and covariates, and the estimated coefficient ( 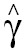) is the slope of true against surrogate measures. In the simplest case, when only outcome, true, and surrogate exposures are considered, the corrected estimate of risk is given by 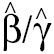, and the variance of the corrected estimate is given by


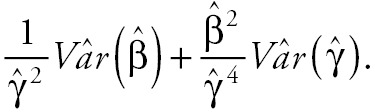


Rosner et al. (1990) developed computational details for models with multiple covariates measured with and without error. In the multivariate version of this method, all covariates included in the primary regression model are included in the measurement error model. Otherwise, the measurement-error–corrected RR estimates will be biased (Rosner et al. 1990). Methods for validating prediction models are not applicable in this context.

From among the list of possible determinants of wheeze considered by Park et al. (2001a) in this main study population, history of lower respiratory infection and race/ethnicity were identified as statistically significant independent risk factors. Park et al. (2001b) also examined the relationship between airborne and house-dust endotoxin and developed a prediction model for airborne endotoxin using internal cross-validation by minimizing the predicted residual error sum of squares (PRESS statistic) (Allen 1971; Svendsgaard et al. 1997). The final model identified current and former presence of a dog in the home, use of a dehumidifier, the total amount of fine dust, and the presence of a concrete floor and water damage as playing key roles in the relationship between airborne and settled-dust endotoxin (Park et al. 2001b).

To apply the regression calibration method of Rosner et al. (1989), we first constructed a main study model that relates the log-transformed probability of experiencing any/repeated wheeze to terms for dust endotoxin level (log_10_-transformed), and covariates using the significant independent predictors identified by Park et al. (2001a, 2001b), as described above. To meet the requirements for this regression calibration method, we also included in the main study model all covariates in the measurement error model. We next constructed a measurement error model that relates the airborne endotoxin level (log_10_-transformed) to the family-room dust endotoxin level (log_10_-transformed), and the covariates identified by Park et al. (2001a, 2001b), as described above. In the same way as for the main model, to apply the Rosner et al. (1989) method, we included the covariates from the main study model in the measurement error model.

In fitting the RR model in the main study and measurement error model in the validation study, when no information was available for a binary covariate, missingness indicators were included. However, for some covariates there were too few missing values to allow for estimation of a missingness indicator; thus, only homes with complete covariate information for these variables were considered eligible for the present study.

The main study model was fit among the main study participants to obtain 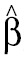, as well as parameter estimates for the other covariates in the model, and the measurement error model was fit in the validation data to obtain 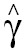 and other parameter estimates.

Before correcting 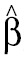 for bias due to measurement error through regression calibration, it is necessary to verify the assumptions required for valid application of this methodology: that *a*) the measurement error model is linear and homoscedastic, *b*) that the main study model is linear on the log prevalence scale, *c*) that house-dust endotoxin is a proper surrogate for the true exposure, and *d*) that the measurement error is not severe.

To assess the linearity of the main study and measurement error models, we compared linear models with more flexible restricted cubic spline models that allow for nonlinearity in the relationship between dust endotoxin level and outcome (Durrleman and Simon 1989). Linearity of the relationship between airborne and dust endotoxin in the measurement error model was confirmed, because none of a wide range of nonlinear terms from a very general model was selected at the *p* = 0.05 level in a stepwise selection procedure. However, there was evidence against a linear relationship between dust endotoxin and the risk of wheeze in the main study (*p* = 0.005, test for nonlinearity). Examination of plots of the fitted spline models suggested linearity might hold on the subset of homes with nonextreme dust endotoxin levels. On refitting the spline models among the observations with dust endotoxin levels between the 5th and 95th percentiles, linearity was confirmed because none of a wide range of nonlinear terms from a 10-knot restricted cubic spline model was selected at the *p* = 0.05 level in a stepwise selection procedure. Thus, only the 442 homes with dust endotoxin measurements above the 5th and below the 95th percentiles were included in the present analysis, leaving *n*_1_ = 360 in the main study and *n*_2_ = 82 in the validation study.

Homoscedasticity of the measurement error was verified by computing the correlation between the fitted values and the squared residuals. After removal of two influential points, as well as the observations with incomplete covariate information, the estimated Pearson’s correlation coefficient between the fitted values and squared residuals was low (*r* = 0.16), indicating that the assumption of homoscedasticity of the measurement error model is reasonable here.

The third assumption requires that once airborne endotoxin exposure is taken into account, house-dust endotoxin has no further independent association with wheeze. We assessed this assumption by comparing two models fit to data from those homes with airborne endotoxin measured concurrently with house-dust endotoxin obtained at the first home visit. The first modeled the relationship between wheeze and airborne endotoxin, and the second included an additional term for house-dust endotoxin. Comparison of these two models did not suggest that the surrogacy assumption was violated—that is, adding dust endotoxin to the model did not produce a significant increase in explanatory power after adjusting for race and lower respiratory infection (*p* = 0.46). Power was low in the data for assessing the consistency of the data with this assumption, because the internal validation study contained only 34 participants with known outcome, of whom nine were cases.

The fourth assumption was examined by computing the multiple correlation coefficient for the measurement error model, as the 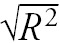. Here, this was 0.67, well within the range of a moderate error scenario needed for valid application of the regression calibration method.

Software to execute these techniques is readily available in the form of SAS macros (SAS Institute Inc., Cary, NC) that can be downloaded from the website of the senior author of this article (www.hsph.harvard.edu/facres/spglmn.html).

## Results

**Figure 1:**
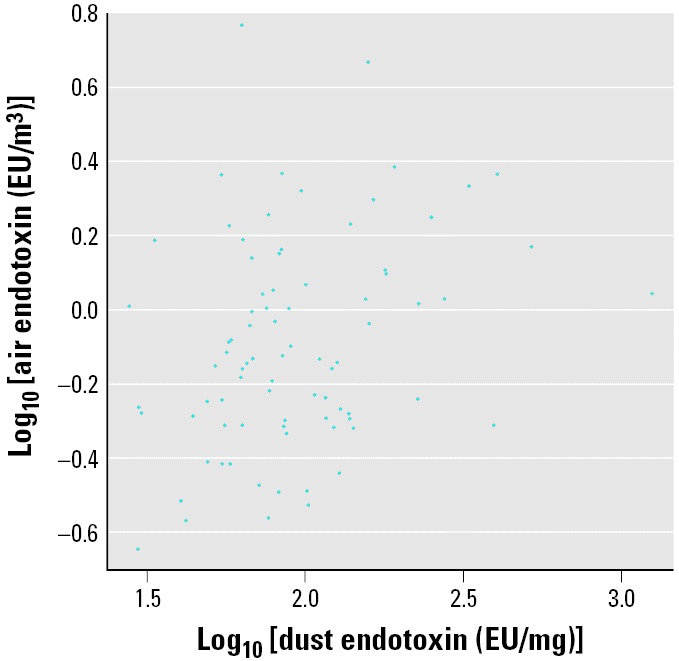
Scatter plot of airborne endotoxin versus dust endotoxin in validation study (*n*_2_ = 82); *r* = 0.29.

Table 1 displays the prevalence of wheeze, information regarding the distribution of endotoxin measurements, and other covariate information. At least one episode of wheeze during the first year of life was reported for 42% of the main study participants included in the analysis. The mean and standard deviation of dust endotoxin levels were very similar in the main and validation study participants, although the range was wider in the validation study. The distribution of other factors is reasonably similar between the two study populations. Figure 1 shows the scatter plot of airborne on dust endotoxin in the validation study data. The estimated correlation coefficient between the true and surrogate exposures on the log scale was 0.29. Estimated coefficients from the measurement error model in the validation study are given in Table 2. The multiple correlation coefficient of the true exposure with the surrogate exposure and other covariates ( 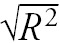) for the fitted measurement error model was 0.67, reflecting considerable ability to estimate true exposure given the surrogate and other variables.

Results of the uncorrected and measurement-error–corrected analyses, in the subset of homes with dust endotoxin measurements above the 5th and below the 95th percentiles, are presented in Table 3. Note that the uncorrected and corrected estimates of RR must be reported on different scales. The uncorrected estimate represents the relative increase in risk associated with an increase over the inter-quartile range in dust endotoxin exposure [0.34 log_10_(EU/mg)], whereas the corrected RR is for an interquartile range increase in air-borne endotoxin exposure of 0.39 log_10_ (EU/m^3^). The univariate analysis revealed substantial deattenuation of risk after correction for measurement error. The uncorrected estimate of RR was 1.33 for a one interquartile range increase in airborne endotoxin (95% CI, 1.11–1.60), but after correcting for measurement error, the estimate of RR was 3.11 for a one interquartile range increase in airborne endotoxin (95% CI, 1.04–9.28). In the multivariate analysis, there was a larger effect of measurement error correction. The uncorrected RR was 1.45 (95% CI, 1.20–1.76), and RR increased to 5.56 (95 CI, 1.19–26.0) after correction for measurement error.

The increase in the width of the CIs associated with the corrected estimates of RR points to three facts: First, with a binary response, the variance increases with the value of the point estimate—as the risk ratio increases, so does its variance. Second, the corrected estimator has an additional component of variation, owing to the uncertainty in the values of the parameters 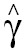 and other coefficients in the measurement error model. Finally, there is an increase in the underlying variability of the estimator due intrinsically to measurement error itself. This is evident in the first term of the function for the variance of the corrected estimate, in which (in the univariate case), the variance of 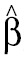 is divided by 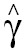


To assess whether an individual’s history of lower respiratory infection acts as an intermediate variable in the relationship between endotoxin exposure and risk of developing wheeze, a measurement error correction analysis was performed on a multivariate model from which the lower respiratory infection covariate was omitted. If this covariate were an intermediate variable rather than a confounder, one would expect the estimate of RR from a model that does not include lower respiratory infection status to increase compared with the estimate that arises from a model including this covariate (Lin et al. 1997). However, because the estimated RR from the analysis in which lower respiratory infection is excluded was less than the estimate obtained in the analysis that controlled for lower respiratory infection, it appeared that lower respiratory infection is more likely to be a confounding variable rather than an intermediate variable and should therefore be adjusted for in multivariate analysis.

## Discussion

This analysis suggests that the prevalence of “any wheeze’“ in the first year of life increases 6-fold for every 0.4 log_10_(EU/m^3^) increase in airborne endotoxin exposure. When house-dust measurements are used to quantify endotoxin exposure, findings have been much more modest. Park et al. (2001a), using a dichotomous exposure variable, reported a 33% increase in “any wheeze” for children exposed to “high” levels of endotoxin in house dust compared with children in “low” exposure households. In the present study, using a continuous exposure variable, the uncorrected risk ratio is 1.45, suggesting a 45% increase in any wheeze per 0.34 log_10_(EU/mg) increase in endotoxin measured in house dust. Clearly, correction for measurement error has a large impact on the point estimate of the effect of increased exposure to endotoxin, underscoring the importance of this substance in inducing or exacerbating wheezing episodes among infants.

One implication of finding a much stronger association of wheeze with airborne endotoxin than with dust endotoxin in the family room is that it is reasonable to assume that airborne endotoxin measured over 1.5 days is a more direct measure of exposure than is dust endotoxin. Dust endotoxin, used to gauge exposure in most epidemiologic studies of domestic exposure and asthma risk, may be a direct exposure measure if the sample is taken from bedding and if the major route of exposure is via large particles inhaled from the bedding surfaces. Alternatively, dust in the family room is necessarily a surrogate for “true” exposure, either from bedding or via an airborne route. A long-term or often-repeated personal breathing zone air sample would be a more precise measure of inhaled endotoxin than anything that is feasible in a large community-based study. For the present analyses, we have assumed that airborne endotoxin is the “true” exposure and that average airborne exposure in the first months of life can be validly estimated by a single measurement collected for an average of 1.5 days. We previously reported, from a small convenience sample of homes in metropolitan Boston, that both floor-dust and air measurements have high within-home variation relative to between-home variation (Park et al. 2000). However, recent analysis of dust endotoxin from the much larger birth cohort described here shows much more favorable ratios of within-home to between-home variance (Abraham et al. 2005). Unfortunately, we do not have repeated measurements of airborne endotoxin in this study. But these data show that single measures of airborne endotoxin taken over 1.5 days were able to identify important differences in exposure between homes.

Another implication of these results is that low-level exposures to endotoxin may have a stronger modulating effect on airway inflammation in young children than previously appreciated. Few birth cohort studies have examined airborne endotoxin in homes. This analysis suggests that airborne measurements may be important in identifying the true magnitude of effects of microbial stimuli to the innate immune system and should be considered in future studies of domestic exposure to endotoxin, peptidoglycan, and other pathogen-associated molecular patterns implicated by the hygiene hypotheses of allergy and asthma pathogenesis (Eder and von Mutius 2004; van Strien et al. 2004).

Several key assumptions need to be met for valid application of regression calibration. First, the measurement error model assumed and fit in the validation study is assumed to apply to the main study as well. Additionally, the measurement error model must be linear and homoscedastic, the main study model must be linear on the log prevalence scale, and the usual exposure measure must contain no further information about the distribution of the outcome when data on the gold standard are available. Each of these assumptions but the first is empirically verifiable (see “Results”). As always in regression analyses when continuous covariates are included, care must be taken to ensure that outliers, or sparse data at the extremes of exposure, are not overly influential. In the study presented here, linearity of the exposure–response relationship may be the most difficult of these assumptions to meet and could be verified only in data between the 5th and 95th percentiles. Results of this study should not be extrapolated beyond the range of the data included in this analysis. Studies of airborne endotoxin exposure in early life that include families with more data falling at high exposures, such as studies in farming communities, are needed to determine whether the dose–response curve remains linear and if the present results are applicable above the 95th percentile of exposure in the present data.

In the present example, we were not able to examine repeated wheeze because, as previously described by Park et al. (2001a), there is a J-shaped relationship between the RR of repeated wheeze and the surrogate exposure. Because of the complex and opposite relationships between endotoxin exposure and atopic and nonatopic wheeze in older children (Braun-Fahrlander et al. 2002; Eduard et al. 2004), regression calibration may not be useful in analyzing endotoxin exposure and the combination of the two outcomes simply identified as “wheeze.” However, it may be useful in examining the relationship of endotoxin and atopic wheeze or endotoxin and nonatopic wheeze, if these outcomes are shown to have a linear exposure–response relationship. Among infants, we cannot distinguish between atopic and nonatopic wheeze, and these analyses of repeated wheeze were not attempted here.

Because of missing exposure and/or covariate data, 12% of both the main study and validation study participants were not included in the analysis. We assume that this moderate amount of missingness was jointly unrelated to exposure and outcome after controlling for observed covariates, and jointly unrelated to the values of the parameter estimates of the measurement error model after controlling for observed covariates. Then selection bias would have little if any impact on the results of this analysis.

The regression calibration approach to measurement error correction has several features that make its use attractive to environmental health researchers. Provided that a set of reasonable assumptions are met, this technique yields an approximately unbiased estimate of the effect of exposure on disease, with associated standard error estimates that fully account for the true uncertainty inherent in estimating health effects from error-prone exposure data. All available data are used, and the corrected estimate of effect is in units of the exposure of interest, rather than the surrogate. Data from the main and validation studies are combined to produce a unified set of results that are easily interpreted.

A limitation of regression calibration is that it cannot increase the underlying power of a given study design. It can improve the validity of the point estimate by removing bias, but the fact that measurement error is present and that its magnitude must be estimated from the validation study limit the power of the analysis. The significance level of hypothesis tests in most common models will not change (Tosteson and Tsiatis 1988), but confidence limits will broaden as point estimates move away from the null. However, this drawback can be overcome by planning to correct for bias due to exposure measurement error at the design stage of a study. Studies can be augmented to include a validation substudy in a cost-efficient manner (Holcroft and Spiegelman 1999; Spiegelman and Gray 1991).

In summary, the analysis of airborne endotoxin presented here confirms earlier findings that endotoxin exposure in early life has important health implications. It supports the hypothesis that inhalation is the relevant route of exposure. This analysis suggests that although uses of surrogate exposure measures such as dust endotoxin are effective means to identify a role of endotoxin in childhood asthma, it also suggests that the magnitude of endotoxin’s effect may be underestimated by such studies. This may be of secondary importance when hypothesis testing is the only goal, but it is important when the goal is to assess the relative impact of various exposures, or to provide a basis for control strategies and regulations, as is typically the case in environmental epidemiology.

## Figures and Tables

**Table 1:** Basic characteristics of the study populations (%).^a^

Characteristic	Main study (*n*_1_ = 360)	Validation study (*n*_2_ = 82)
Any wheeze (≥ 1 episode)	42	26
Lower respiratory illness (≥ 1 episode)	28	21
Race/ethnicity		
White	78	81
Black	11	13
Hispanic	6	0
Asian	4	4
Other	1	2
Presence of dog		
Current	17	20
Former	21	23
Use of dehumidifier	20	17
Presence of concrete floor	7	5
Presence of water damage	36	41
Dust endotoxin (EU/mg) [mean (minimum–maximum)]	79.6 (26.2–241.6)	93.1 (27.7–1249.0)
Airborne endotoxin (EU/m^3^) [mean (minimum–maximum)]	—	0.81 (0.23–5.87)
Total fine dust (g) [mean (minimum–maximum)]	1601.6 (258.0–11467.0)	1329.1 (477.0–6075.0)

a Values are percentages unless noted otherwise.

**Table 2:** Measurement error model for log10(airborne endotoxin) [log_10_(EU/m^3^)] (*n*_2_ = 82).

Variable	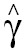	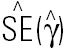	*p*-Value
Log_10_(dust endotoxin) [log_10_(EU/mg)]	0.25	0.09	< 0.01
Log_10_(total fine dust) [log_10_(g)]	0.21	0.11	0.05
Lower respiratory infection	0.07	0.06	0.30
Race/ethnicity			
Black	0.04	0.08	0.64
Asian/other^a^	0.18	0.11	0.11
Presence of dog			
Current	0.22	0.07	< 0.01
Former	0.14	0.07	0.03
Use of dehumidifier	−0.11	0.08	0.15
Presence of concrete floor			
Living room	0.28	0.12	0.03
Dining room and kitchen	0.28	0.15	0.06
Presence of water damage	0.11	0.05	0.04

a There are no Hispanics in the validation study.

**Table 3:** Association between endotoxin exposure and wheeze (*n*_1_ = 360, *n*_2_ = 82).

	Uncorrected		Corrected	
Model	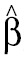 (*p*-value)	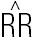 a (95% CI)	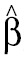 (*p*-value)	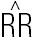 b (95% CI)
Univariate	0.84 (< 0.01)	1.33 (1.11–1.60)	2.91 (0.04)	3.11 (1.04–9.28)
Multivariate^c^	0.89 (< 0.01)	1.35 (1.11–1.65)	3.63 (0.05)	4.12 (1.01–16.83)
Multivariate^d^	1.09 (< 0.01)	1.45 (1.20–1.76)	4.40 (0.03)	5.56 (1.19–26.03)

a Estimated RR reflects an increase of one interquartile range [0.34 log10(EU/mg)] in dust endotoxin exposure.

b Estimated RR reflects an increase of one interquartile range [0.39 log_10_(EU/m^3^)] in airborne endotoxin exposure.

c Adjusted for race, presence of dog in home, former (not current) dog in home, use of dehumidifier, total mass of dust sample collected (in log scale), presence of concrete floor, missingness indicator for presence of concrete floor, and presence of water damage in the measurement error model.

d Further adjusted for lower respiratory illness, in addition to covariates of the previous multivariate model.

## Appendix 1. Derivation of the regression calibration estimator for a relative risk (log-binomial) model

Assume *Pr* (*Y*=1|*X*, **U**) = e^β0 +^ *^X^*^β1 +^ ***^U^***^β^**2**, where *Y* is a binary outcome, *X* is the true exposure variable, *Z* is a surrogate for *X*, and **U** is a vector of covariates assumed perfectly measured. Note that the relative risk equals e^β_1_^, where Δ is an increment in *X* of biological or public health significance. Further, assume that μ*_X_*_|_*_Z_*_,_**U** = *E*(*X*|*Z*,**U** ) = α_0_ + *Z*α_1_ + **U**α**2** and *Var*(*X*|*Z*, **U**) = α^2^*_X_*_|_*_Z_*_,_**U**_._

### Integrate directly.

If the assumptions about the measurement error model are appropriate, *Pr*(*Y*=1|*Z*,**U**) = ∫*_x_* *exp*(β_0_ + *X*β_1_ + **U**β**2**) *f**_X_*_|_*_Z_*_,_**U**(*X*|*Z*,**U** ) *dx*, where *f**_X_*_|_*_Z_*_,_**U** is the normal density function of *X* given (*Z*,**U**) with mean μ*_X_*_|_*_Z_*_,_**U** and variance σ^2^*_X_*_|_*_Z_*_,_**U**. After some algebra and completing the square, *Pr*(*Y*=1|*Z*,**U**) = *exp*(β_0_ + 1/2β_1_^2^ σ^2^*_X_*_|_*_Z_*_,_**U** + μ*X* |*Z*,**U** β_1_ + **U**β**2**). If the measurement error model fits the data, then model for the probability of the disease outcome given *Z* and **U** is given by *Pr* (*Y*=1|*Z*,**U**) = *exp*(α_0_ + *Z*α_1_ + **U**α_2_), where α_0_ = β_0_ + 1/2β1^2^σ^2^*_X_*_|_*_Z_*_,_**U** + γ_0_β_1_, α_1_ = β_1_γ_1_ and α_2_ = β_1_γ**2** + β_2_.

### Taylor series expansion.

An approximation of *Pr* (*Y*=1|*Z*,**U**) can be found without the normality assumption for *f* *_X_*_|_*_Z_*_,_**U** by substituting the second-order Taylor series expansion of *Pr* (*Y*=1|*X*,**U**) about μ*_X_*_|_*_Z_*_,_**U** in *Pr* (*Y*=1|*Z*,**U** ) = ∫ *Pr* (*Y*=1|*X*,**U**) *f**_X_*_|_*_Z_*_,_**U** (*X* |*Z*,**U** )*dx*. After taking the expectation of the expansion, the linear term is equal to zero. The following approximation is found:

If β_1_^2^ σ^2^*_X_*_|_*_Z_*_,_**U** is close to zero, the third term disappears and *Pr* (*Y*=1|*Z*,**U** ) is approximated by exp[β_0_ + β_1μ_*_X_*_|_*_Z_*_,_**U** + **U**β**2**].

Regardless of which derivation (and which assumptions) are used, if estimates of α′ =(α_0_, α_1_, α_2_′) are obtained from the model of the probability of disease in relation to *Z* and **U** and estimates of γ′ = (γ_0_, γ_1_, γ_2_′) are obtained from the measurement error model of *X* on *Z* and **U**, an estimated exposure effect corrected for measurement error is computed by . The measurement error-corrected effects of the perfectly measured covariates are estimated by . The measurement error-corrected intercept is estimated by  when β_1_^2^σ^2^*_X_*_|_*_Z_*_,_**U** ≡ 0.

The variance of these estimates is derived from the multivariate delta method.
